# Misconceptions of pathophysiology of happy hypoxemia and implications for management of COVID-19

**DOI:** 10.1186/s12931-020-01520-y

**Published:** 2020-09-24

**Authors:** Martin J. Tobin, Amal Jubran, Franco Laghi

**Affiliations:** Division of Pulmonary and Critical Care Medicine, Hines Veterans Affairs Hospital and Loyola University of Chicago Stritch School of Medicine, Hines, IL 60141 USA

## Abstract

In the article “The pathophysiology of ‘happy’ hypoxemia in COVID-19,” Dhont et al. (Respir Res 21:198, 2020) discuss pathophysiological mechanisms that may be responsible for the absence of dyspnea in patients with COVID-19 who exhibit severe hypoxemia. The authors review well-known mechanisms that contribute to development of hypoxemia in patients with pneumonia, but are less clear as to why patients should be free of respiratory discomfort despite arterial oxygen levels commonly regarded as life threatening. The authors propose a number of therapeutic measures for patients with COVID-19 and happy hypoxemia; we believe readers should be alerted to problems with the authors’ interpretations and recommendations.

**Letter**

We read with interest “The pathophysiology of ‘happy’ hypoxemia in COVID-19” by Dhont et al. [1]. We agree with many of their points but disagree on several important facets.

Dhont and colleagues [1] claim that increases in respiratory rate and tidal volume are “the most important clinical signs of impending hypoxemic respiratory failure.” On the contrary, neither rate nor tidal volume are sensitive or specific for hypoxemia. The essential point about happy hypoxemia is that patients can be profoundly hypoxic and yet exhibit no abnormality in breathing pattern [2].

Dhont et al. [1] claim that a leftward shift in the oxyhemoglobin-dissociation curve explains “why SpO_2_ can be well-preserved in the face of a profoundly low PaO_2_.” Given that the carotid bodies respond solely to arterial oxygen tension (PaO_2_), and not to arterial oxygen saturation (SaO_2_) [1], a leftward shift of the dissociation curve would increase the likelihood of dyspnea—the opposite of happy hypoxemia.

Diagnosis of happy hypoxemia in COVID-19 is typically prompted by low pulse oximeter readings. Pulse oximetry markedly exaggerates the severity of low oxygen saturations when readings are low [2] (and this will not enhance carotid-body stimulation). Additionally, fever (a frequent occurrence in COVID-19) moves the oxygen-dissociation curve to the right. For example, a temperature of 40 °C will produce a decrease in oxygen saturation of 9.9% without change in PaO_2_ [2]. This substantial desaturation will not increase carotid-body stimulation—a perfect set-up for happy hypoxemia.

Dhont and colleagues [1] claim that increases in negative inspiratory intrathoracic pressure in COVID-19 patients will produce patient self-inflicted lung injury (P-SILI). There is no evidence that P-SILI occurs in patients with COVID-19 [3]. Indeed, there is no direct experimental proof for the occurrence of P-SILI in human subjects [4].

Dhont et al. [1] invoke involvement of pulmonary vasculature and intravascular microthrombi in COVID-19. There is, however, no known physiological mechanism whereby such involvement can cause suppression of dyspnea—the clinical hallmark of happy hypoxemia [2].

Dhont and colleagues [1] claim that reduction of cytokine storm is a major therapeutic goal in COVID-19. Where are the data to indicate that specific measures ameliorate cytokine storm and where is the evidence that measures directed at such a target will benefit patients?

The authors list a series of therapies—including tissue plasminogen activator (tPA), anti-inflammatory agents (tocilizumab, sarilumab, siltuximab), and modulators of the renin-angiotensin system—for use in COVID-19 patients. Each agent carries major potential for patient harm, and there is no conceivable mechanistic pathway whereby they will reverse the absence of dyspnea in hypoxemic COVID-19 patients (happy hypoxemia) [2].

Dhont and colleagues [1] claim that intubation and invasive ventilation is advantageous over non-invasive ventilation through decreases in oxygen debt, by avoidance of P-SILI, and by offering a better chance for the lungs to heal. No form of ventilator support is motivated by concerns about oxygen debt [5]. There is no proof that P-SILI occurs in COVID-19 patients [3, 4]. No form of ventilator support has been shown to increase lung healing [5].

Dhont et al. [1] claim that most severely ill COVID-19 patients fulfil the Berlin criteria of ARDS, and on this basis judge certain aspects of mechanical ventilation as “key” steps. Problems that ensue from such a mindset is highlighted by data contained in a report from a NIH-ARDS Network center, which involved 66 patients with COVID-19 (85% with ARDS) who were “managed with mechanical ventilation and established ARDS protocols” [6].

Analyzing these data, Yaroshetskiy et al. [7] point out that the patients had relatively high PaO_2_/FiO_2_ (median 245 [equivalent to PaO_2_ 98 mmHg with fractional inspired oxygen concentration, FiO_2_, of 40%], and PaO_2_/FiO_2_ 320–560 in many); plateau pressure of only 21 cmH_2_O; driving pressure of only 11 cmH_2_O; positive end-expiratory pressure (PEEP) higher when prone than when supine; administration of paralytic agents in 42%; and administration of vasopressors in 95% [6]. Based on the reported physiological variables, Yaroshetskiy et al. [7] ask a rhetorical question “Do all these patients definitely require intubation and mechanical ventilation?”

For the clinician at the bedside of a COVID-19 patient, the only consequent of making a diagnosis of ARDS is avoidance of tidal volume 12 ml/kg. Given that tidal volume 12 ml/kg is not employed in any patient, making a diagnosis of ARDS does not impact selection of any ventilator setting in COVID-19 [8].

We are concerned that Dhont and colleagues [1] link the phenomenon of happy hypoxemia to drastic therapies of unproven benefit and such therapies are more likely to be harmful than hypoxemia—which frequently responds to much simpler measures [9].

## Data Availability

Not applicable.

## Abbreviations

COVID-19: Coronavirus Disease 2019
PaO_2_: Arterial oxygen tension
SaO_2_: Arterial oxygen saturation
P-SILI: Patient self-induced lung injury
tPA: Tissue plasminogen activator
ARDS: Acute respiratory distress syndrome
NIH: National Institute of Health
FiO_2_: Fractional inspired oxygen concentration
PEEP: Positive end-expiratory pressure
